# A tropical tertiary neurosurgical centre response to COVID-19 pandemic and its effect on neurosurgical practices

**DOI:** 10.4314/ahs.v22i3.55

**Published:** 2022-09

**Authors:** Olufemi Idowu, Hammed Oshola, Jeuel Idowu, Ademola Omosuyi

**Affiliations:** 1 Lagos State University College of Medicine, Surgery (Neurosurgery Unit); 2 Lagos State University Teaching Hospital, Surgery (Neurological Surgery Unity)

**Keywords:** COVID-19, Neurosurgery, Protocols, SARS-CoV-2

## Abstract

**Background:**

COVID-19 pandemic may decrease the quantum of care for patients with neurosurgical conditions.

**Objectives:**

To determine outpatient clinic (OPC) patient load, neurosurgical procedures volume and disease spectrum following the institution of a new care protocol during the ongoing COVID-19 pandemic and compare with previous practice data in our institution.

**Methods:**

A monocentric retrospective analysis of all patients requiring neurosurgical care over a 2-year period.

**Results:**

There was a 42.4% reduction in OPC attendance and 41.8% reduction in surgical procedures in 2020 compared to 2019. There was >60 percent reduction in the volume of surgery that was done at the onset and peak of the pandemic but this has normalized in November 2020 despite the resurgence of COVID-19, after the institution of a new care protocol. Neurotrauma procedures (29.6%) were the most common neurosurgical operation in 2020 while congenital malformation surgery (37.3%) was the most common procedure performed in 2019.

**Conclusions:**

The ongoing COVID-19 pandemic initially led to significant decrease in quantum and spectra of patients who presented at the OPC and for neurosurgical procedures. Instituted local protocol and Teleclinics, if added to clinical care armamentarium, may help to improve on the low patient attendance during pandemics.

## Introduction

The World Health Organization (WHO) declared severe acute respiratory syndrome coronavirus 2 (SARS-CoV-2) infection a public health emergency of international concern on the 31^st^ of January 2020 and a pandemic on 11^th^ March 2020.^1^,^2^ Since the declaration, coronavirus infectious disease 2019 (COVID-19) has continued to affect all aspects of the society, including clinical care protocol, patients' volume and disease spectrum in the hospitals, number of surgical procedures and health care delivery as a whole.

Most countries that have vaccinated a sizeable proportion of their citizenry against SARS-CoV-2 are likely to experience reductions in SARS-CoV-2 infection rates. In most low- and middle-income countries (LMICs) there is limited access to SARS-CoV-2 vaccines and substantial vaccination in these countries may not be achieved till 2023.^3^,^4^ Thus, the effect of COVID-19 pandemic will most likely remain a challenge for years to come.

Many surgical subspecialties including neurosurgical services are not immune to the adverse effect of COVID-19 in the form of declining outpatient clinic and surgical load. More so in LMICs where inadequate manpower, infrastructure and facilities still persists in addition to its peculiar cultural and religious beliefs which negates against early hospital presentation.^5^ The various surgical subspecialties which were already challenged in the pre COVID-19 era in LMICs are also confronting the effect of the COVID-19 pandemic scourge. Any decrease in the quantum of care for patients with neurosurgical conditions may significantly increase the incidence of patient morbidity and or mortality.

Physical distancing, use of face masks, strict hand hygiene, high index of suspicion, exhaustive patient travel history and appropriate personal protective equipment (PPE) are some ways to significantly reduce the risk of exposure to SARS-CoV-2. Different specialties have developed specific guidelines in other to be able to continue to provide safe and effective care to patients despite the lingering COVID-19 pandemic. Surgical specialties have had to triage the urgency of their outpatient and surgical procedures where possible. This has considerably impacted practice with different specialties modifying their existing standard operating procedures and guidelines on how to manage their patients.^6^–^8^ These modifications of protocols involve patient and staff safety guidelines, clinic workflow, surgical protocols and medical training. The protocols are continuously revised to maintain an effective level of functioning in the midst of prevailing local deficiencies in facilities, equipment and personnel. Using National and international guidelines, recommendations and position statements, the development of risk stratification, prioritization, and scheduling of neurosurgical procedures have been facilitated.^9^

The aim and objectives of this study is to examine our outpatient clinic (OPC) and neurosurgical operations patient load, demographics and spectrum of diagnosis at our academic tertiary neurosurgical centre while the COVID-19 pandemic continues and to compare the quantum of these variables with the penultimate pre COVID-19 pandemic era. This study will provide some beneficial tips to centres located in other LMICs.

## Material and Methods

This descriptive correlational study was carried out at the Lagos State University Teaching Hospital, Ikeja, Lagos, Nigeria. Nigeria is located in the Gulf of Guinea on the west coast of Africa with an estimated population of 206,140 million and land mass of 923,768 km^2^.^10^ It has a 36-state structure with Lagos State (its smallest state by land mass) accounting for 10 % of Nigeria's population and the epicentre of the COVID-19 pandemic in Nigeria. The study hospital, Lagos State University Teaching Hospital, Ikeja, Lagos, Nigeria, is situated in Lagos state, Nigeria. The hospital is owned by the Lagos state government and was upgraded in 2002 to a University Teaching Hospital status while Neurosurgical operations commenced in December 2004.

### COVID-19 Protocols

Using national and international guidelines, recommendations and position statements, a local COVID-19 protocol was developed and applied locally. As a part of COVID-19 pandemic protocol, patients and staff had noncontact temperature check prior to entering the clinic and operating theatre and within entrances of various sections in the hospital. In-person conferences and seminars were cancelled and replaced with webinars through video teleconferences. Universal precautions including face masks and social distancing were enforced for all group-based activities (ward rounds, etc). Anyone with fever or acute infections is considered to have SARS-CoV-2 until proven otherwise. During consultation at a social distance of at least 2 metres, patients and staff are to wear face masks. All patients been worked up for surgery underwent a preoperative real-time quantitative polymerase chain reaction analysis of their nasopharyngeal swab. A negative report was considered sufficient to proceed for any neurosurgical procedure. The reports were valid for 7days from the time of sample collection. Except in emergencies, all elective and semi urgent surgeries were suspended until a negative COVID-19 test. All patients were required to wear cloth, surgical or N95 mask. Infected patients were only operated at least 2 weeks after COVID-19 symptoms had resolved. Outcome measures The clinical data of all cohort patients (in a single centre) who presented at our outpatient clinic (OPC) and those who underwent major neurosurgical intervention between January 2019 and December 2020 were included in the study. Patients that had minor procedures (i.e. ventricular tap, transforaminal epidural injections, etc) were excluded from the surgical data. Data collected from the OPC and surgical logs included year/month of patient presentation, demography, diagnosis, surgical procedure, indication for surgery and region of the body operated. The generated data were divided into two groups – pre-COVID period (January, 2019- December, 2019) and during the COVID pandemic (January, 2020-December, 2020). There was further subdivision of the groups into various categories (neurotrauma, neoplastic, neurovascular, congenital malformations, infective and others). The primary outcome of the study was the number of new and old patients who presented at the OPC and the number of patients who were operated during the study period. The secondary outcomes were cranial/spinal surgeries change in 2020 compared to 2019.

Qualitative data variables were described using frequency and percentages. The diagnosis, and surgeries performed (cranial or spinal) were compared between the two groups. Differences in categorical variables between groups were compared using Chi-square test (χ2) analysis. Continuous variables are expressed as mean ± standard deviation (SD) and analysed by unpaired t-test and comparison was done between them. A p-value less than 0.05 were considered statistically significant. Data were obtained and analysed with statistical analysis performed with IBM Statistical Product and Service Solutions (SPSS) Statistics for Windows, version 23 (IBM Corp., Armonk, N.Y., USA).

## Results

The demographic and clinical data of patients who attended the outpatient clinic during the study period are summarized in Table 1. There was a 42.4% reduction in OPC attendance in 2020 compared to 2019 (Table 1). The M:F ratio was 1.3:1 in 2019 but 1.2:1 in 2020. There was no statistical significant difference between the gender proportion of patients who attended OPC in 2019 and 2020 (p=0.416). When compared to patients from the pre-COVID-19 era cohort (2019), the COVID-19 era (2020) outpatient clinic patients' cohort had higher proportions of new cases (31%) compared to the pre-COVID-19 era cohort (25.6%).

**Table 1:** Profile of outpatient clinic attendance in 2019 and 2020

Variable	2019(%)	2020(%)	p value
**Total**	2210	1273	
**Gender**			
Male	1257(56.9)	706(55.5)	0.416
Female	953(43.1)	567(44.5)	
**Region**			
New cases	565(25.6)	394(31)	0.0006
Follow up	1645(74.4)	879(69)	
**Diagnosis classification**			
Neurotrauma	556(25.2)	306(24)	0.024
Degenerative spine	446(20.2)	294(23.1)	
Congenital Malformations	444(20.1)	209(16.4)	
Neoplastic	393(17.8)	252(19.8)	
Others	371(16.8)	212(16.7)	

**Figure 1:**
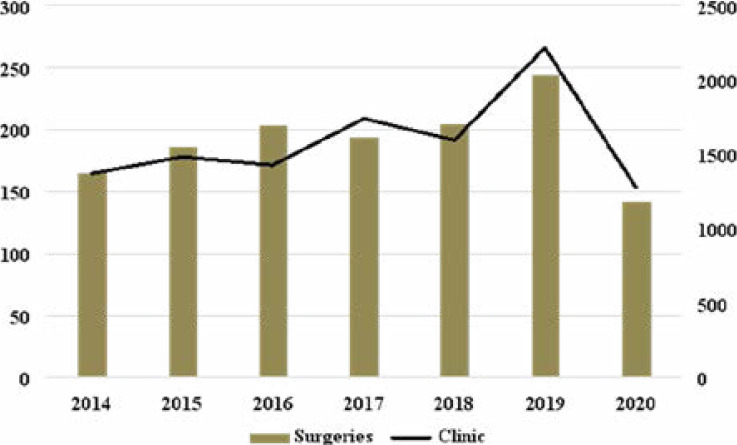
Clinic attendance and surgeries performed over 7 years.

The number of patients who had neurosurgical procedures and attendance OPC from 2014 to 2020 is as depicted in Figure 1. Two hundred and forty-four neurosurgical procedures were done in 2019 compared to 142 in 2020 (41.8% reduction). The surgical patients M:F ratio decreased in 2019 from 1.5:1 to 1.4:1 in 2020. There was no statistical significant difference between the gender proportion of patients who were operated 2019 and 2020 (p=0.858). The mean age of patients operated in 2019 (28.4±3.5years) and 2020 (31.6±4.1years) were similar (p=0.267). The surgical patients' peak age was in the 1st decade of life for patients in 2019 and 2020. Neurotrauma procedures (29.6%) were the most common neurosurgical operation in 2020 (COVID-19 epochs). This was closely followed by surgeries for congenital malformations (23.9%) and tumours (14.8%). In 2019 (pre-COVID-19 era), most of the surgeries performed were due to congenital malformations (37.3%). This was followed by neurotrauma and neoplastic procedures (Table 2).

**Table 2:** Profile of operated patients in 2019 and 2020

Variable	2019(%)	2020(%)	p value
**Age at surgery (years)**			
Mean in years (95% CI)	28.4±3.5	31.6±4.1	0.267
**Gender**			
Male	147(60.2)	84(59.2)	0.833
Female	97(39.8)	58(40.8)	
**Surgery classification**			
Cong Mal	91(37.3)	34(23.9)	0.002
Trauma	80(32.8)	42(29.6)	
Neoplastic	35(14.3)	21(14.8)	
Infection	20(8.2)	18(12.7)	
Degenerative	14(5.7)	15(10.6)	
Neurovascular	4(1.6)	12(8.5)	
**Surgery region**			
Cranial	199(81.6)	105(73.9)	0.078
Spinal	45(18.4)	37(26.1)	

**Figure 2:**
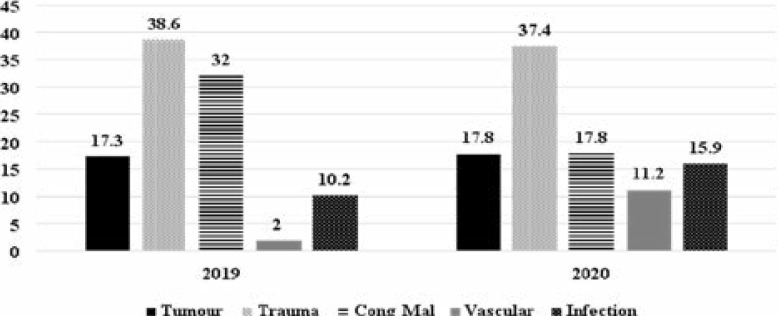
The proportion of various cranial surgeries performed in 2019 and 2020.

Most spinal surgeries in 2019 (28, 59.6%) and 2020 (15, 42.9%) were due to congenital malformations. This was closely followed by degenerative spine disease procedures of 14 (29.8%) in 2019 and 15 (42.9%) in 2020. Cranial surgeries accounted for 81.6% of surgeries done in 2019 as compared with 73.9% of surgeries done in 2020; this was not a statistically significant decrease (p=0.078). The types of surgeries done during the study period are depicted in figure 2.

Three health workers contracted mild COVID-19 from patients during the study period, prior to the institution of the hospital protocol. There was no case of transmission of the disease in our unit, since the enforcement of the protocol.

## Discussion

The first confirmed case of COVID-19 in Nigeria was in Lagos state, on the 27^th^ of February 2020.^11^ This led to a nationwide lockdown on 30th March 2020 after which a phased and this was eased on 4^th^ May 2020. The peak of the outbreak was between June and July, with a second wave in December 2020.^12^ Till date, Lagos state remains the epicentre of the disease in Nigeria, accounting for over 70% of cases in Nigeria.

Nigeria is like most other developing countries with limited human and modern technology for sustained optimal neurosurgical practice. Though the role of neurosurgical management of various diseases has continued to increase (Figure 1), the pandemic is an additional challenge to neurosurgical practice in Nigeria. Health workers and patients are susceptible to contracting the disease in the hospital environment in addition to in the community. Hence, the need to develop and ensure local protocol so as to prevent the spread of the disease within the hospital that is involved in treating COVID-19 patients.

Our institutional neurosurgical care protocol was modified regarding patient screening and triaging. Physical distancing, regular hand washing and hygiene, use of face masks, and use of appropriate personal protective equipment were strictly followed by our health care workers to minimize exposure. What we instituted has helped a long way to militate against contracting the disease as there has been only 3 cases of patient to health worker transmission of the disease in our unit, since the enforcement of the protocol. Teleclinic was attempted at the outpatient clinic but this was not successful despite multiple efforts due to local patient challenges. This was accounted for mainly due to limited and ineffective broadband internet access and poor internet connectivity.

The scourge of this global pandemic will probably persist for many more years to come, especially in the tropics. Neurosurgeons in Africa are facing a unique set of challenges, including, prioritization of brain and spinal cases while confronting different ethical dilemmas in the face of limited resources. Neurosurgical cases are been suspended or postponed. Even though some OPC attendance and surgeries can be deferred for a period of time in different clinical scenarios, postponement of appropriate medical and or surgical treatment may lead to permanent neurological deficits and a substantial decline in the quality of life, or even death. Although less common than most procedures we did, urgent open intracranial vascular surgeries tripled in number in 2020 (Figure 2). The vascular procedures were mainly for ruptured intracranial aneurysms (67%). Other vascular procedures done were for excision of ruptured arteriovenous malformations and cavernomas. The increase in vascular cases we suspect was due to relative increase in hospital bed space for patients with critical conditions ensuing from total decrease in hospital admissions emanating from non-presentation by patients due to fear of contracting SARS-Cov-2.

The impact of the COVID-19 pandemic on different aspects of the neurosurgical service at our academic centre was similar to several experiences reported at other institutions. At the Neurosurgery Teaching Hospital in Baghdad, Iraq, Hoz et al.,^13^ reported a net decrease of 67% of the operative neurosurgical cases from January to April 2020 when compared to the same period in 2019. Similarly, Goyal et al.^14^ in India reported a 52.2% reduction in the number of neurosurgeries performed during the COVID pandemic as compared to the pre-COVID era. In the publication by Burks et al.,^15^ at the Department of Neurological Surgery, University of Miami Miller School of Medicine, 66% and 50% fewer cases performed as compared to the year prior in April and May 2020 respectively while at Emory University Hospital, an 80% reduction in neurosurgical case volume was reported.^16^ Jean et al.,^17^ noted that 52.5% of elective cases and clinics had been shut down by hospital policy, with 46.1% reporting that their operative volume had dropped more than 50%. For the COVID-19 ravaged countries; this proportion was even higher, at 54.7%.^17^ By reducing surgical OPC attendance, surgical procedures and affecting health workers, COVID-19 has a possible deleterious implications for patient care and trainee progression.

**Figure 3:**
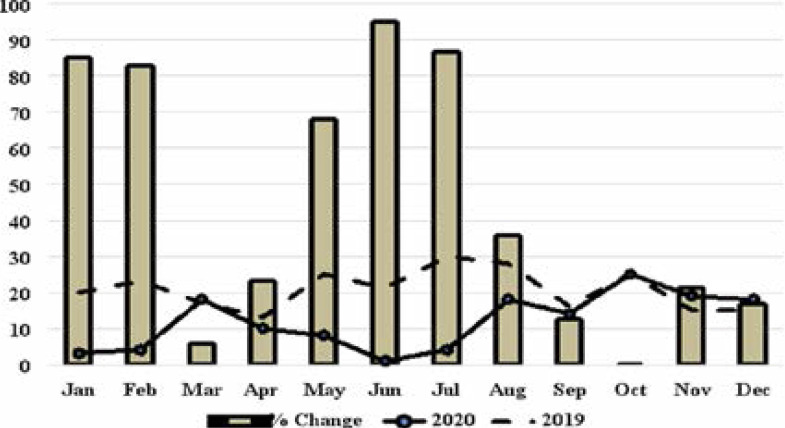
The number and percentage change of surgical cases per month in 2019 and 2020.

Our data shows that >60 percent reduction in the volume of surgery that was done at the onset and peak of the pandemic during the first wave in Lagos (Figure 3). In this study, we found significantly higher reduction of surgical cases and OPC attendance during the peak of the COVID-19 outbreak in Lagos (June-July 2020) when compared with the same period in 2019 (June-July) (Figure 3). This is despite the lockdown being lifted a month earlier. In addition, there was ≥60% reduction in the number of patients in the 1^st^ and 8^th^ decade of life that had surgery in 2020 when compared with similar period in 2019. This reduction in the number of patients attending OPC clinic and number of surgical procedures improved substantially despite the continuity of the pandemic and a second wave (Figure 3). Our study shows the ability to maintain adequate neurosurgical care and improvement in patient case load during a pandemic by applying appropriate standard protocols.

## Limitation

The retrospective nature of the study, while the accuracy of records extraction from our archives can be limited by suboptimal data input.

## Conclusion

This study demonstrated that the ongoing COVID-19 led to significant decrease in the quantum and spectra of neurosurgical OPC patient load and neurosurgical procedures volume when compared with the same period in 2019. Delivery of safe care can be well maintained with proper execution of local protocols. Instituted local protocol helped to militate against patient-health worker SARS-CoV-2 infection spread and thus its beneficial implications for patients' care and trainee progression. The extent to which the current COVID-19 pandemic affects Neurosurgical practice can help health care professionals prepare for similar events in the future. Teleclinics, if added to clinical care armamentarium, may help to improve on the low patient attendance.

## Abbreviations

COVID-19: Coronavirus infectious disease 2019
OPC: Outpatient clinic
LMICs: In most low- and middle-income countries
SARS-CoV-2: Severe acute respiratory syndrome coronavirus 2
WHO: World Health Organization
